# Supplementary material for: HIV-1 Infection of Long-Lived Hematopoietic Precursors In Vitro and In Vivo
Source: Cells. 2022 Sep 23;11(19):2968. doi: 10.3390/cells11192968 (PMC9562211; doi:10.3390/cells11192968)
Supplement: Supplementary file 1 [file cells-11-02968-s001.zip › cells-1883877-supplementary.pdf]

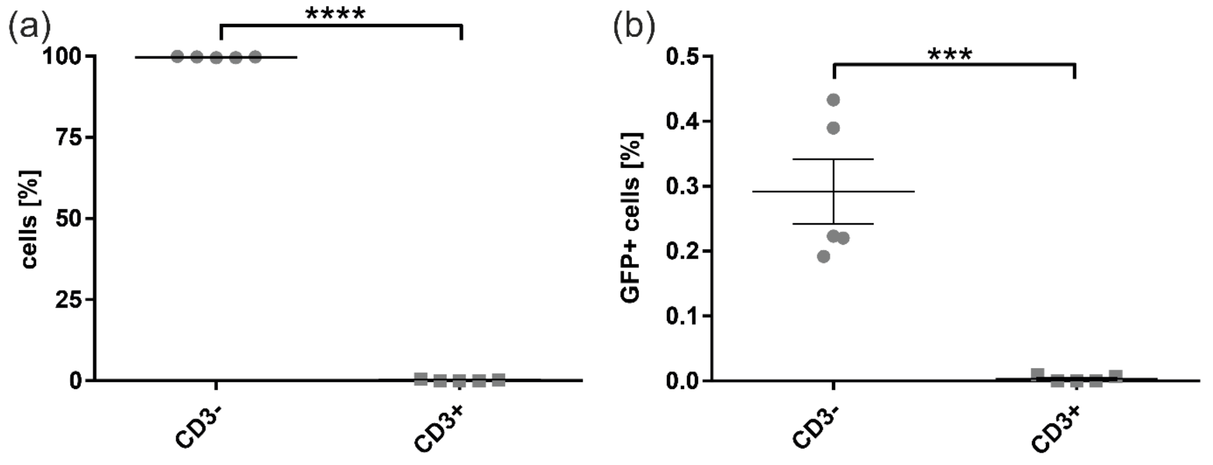

average purity CD3- cells among Lin- cells: 99,56%

average purity CD3- cells among Lin-GFP+ cells: 98,21%

Figure S1. Purity and frequency of CD3+ T cell contaminants in bone marrow derived HSPCs. (**a,b**) Bone marrow derived Lin- cells after two-round lineage depletion (A) were transduced with HxB2 (X4) Env-pseudotyped reporter virus with an MOI of 10 (**b**). The cells were analyzed three days after transduction for CD3 (**a,b**) and GFP expression (**b**). Mean and standard error of the mean are indicated (*** $p < 0.001$; **** $p < 0.0001$). Results of $n = 5$ experiments are shown.

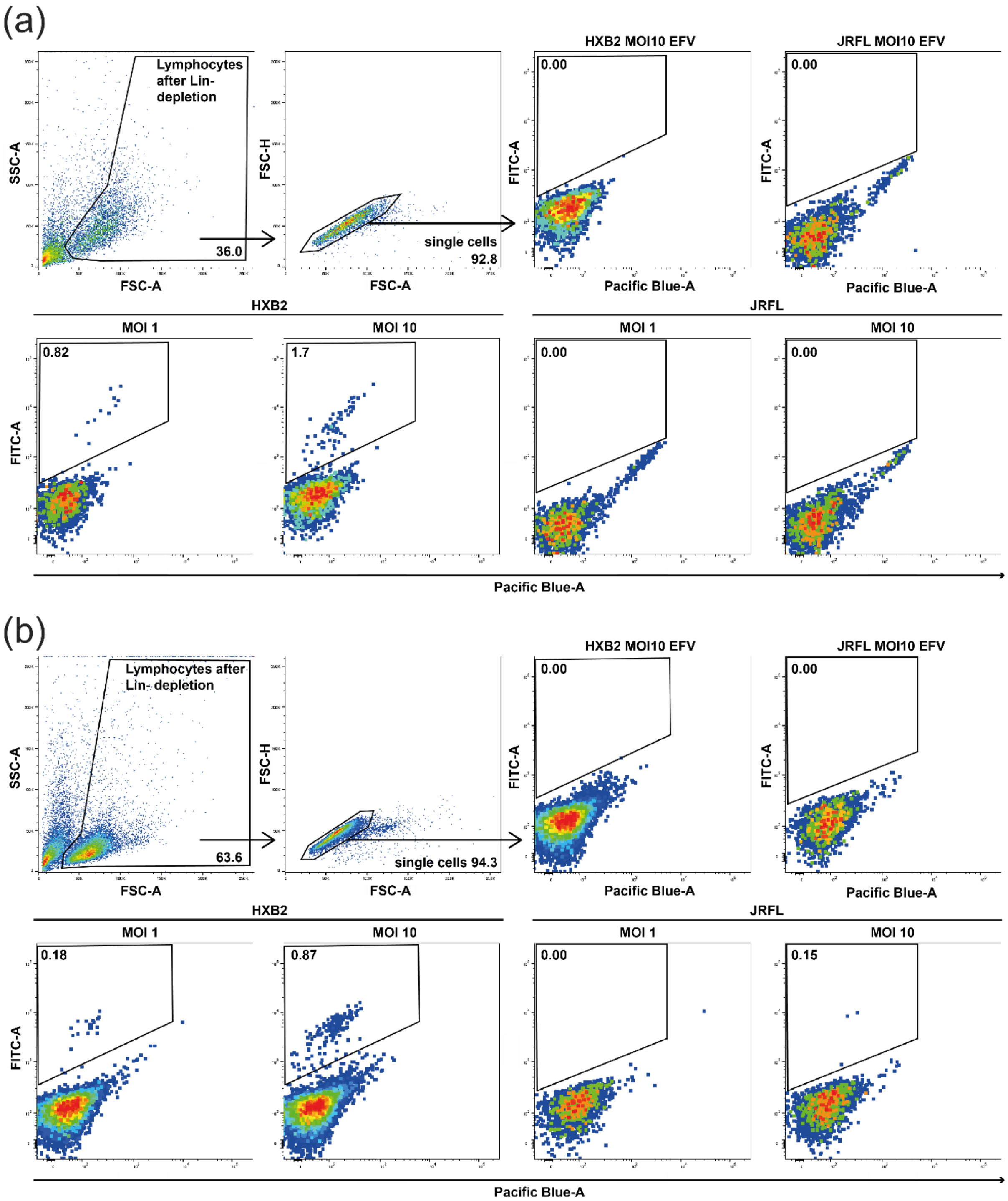

Figure S2. Flow cytometry analysis of in vitro-transduced cord blood and bone marrow-derived HSPCs. (**a**,**b**) Cord blood-derived Lin- cells (**a**) and bone marrow-derived Lin- cells (**b**) were transduced with HxB2 (X4) or JRFL (R5) Env-pseudotyped reporter viruses with an MOI of 1 and 10. The cells were analyzed three days after transduction for GFP expression. Depicted numbers indicate the infection rates. Cells treated with efavirenz (EFV) served as negative control. The Pacific Blue channel was used for gating only (unstained). One example of $n = 6$ (HXB2) and $n = 3$ (JRFL) experiments/donors, respectively, is shown.

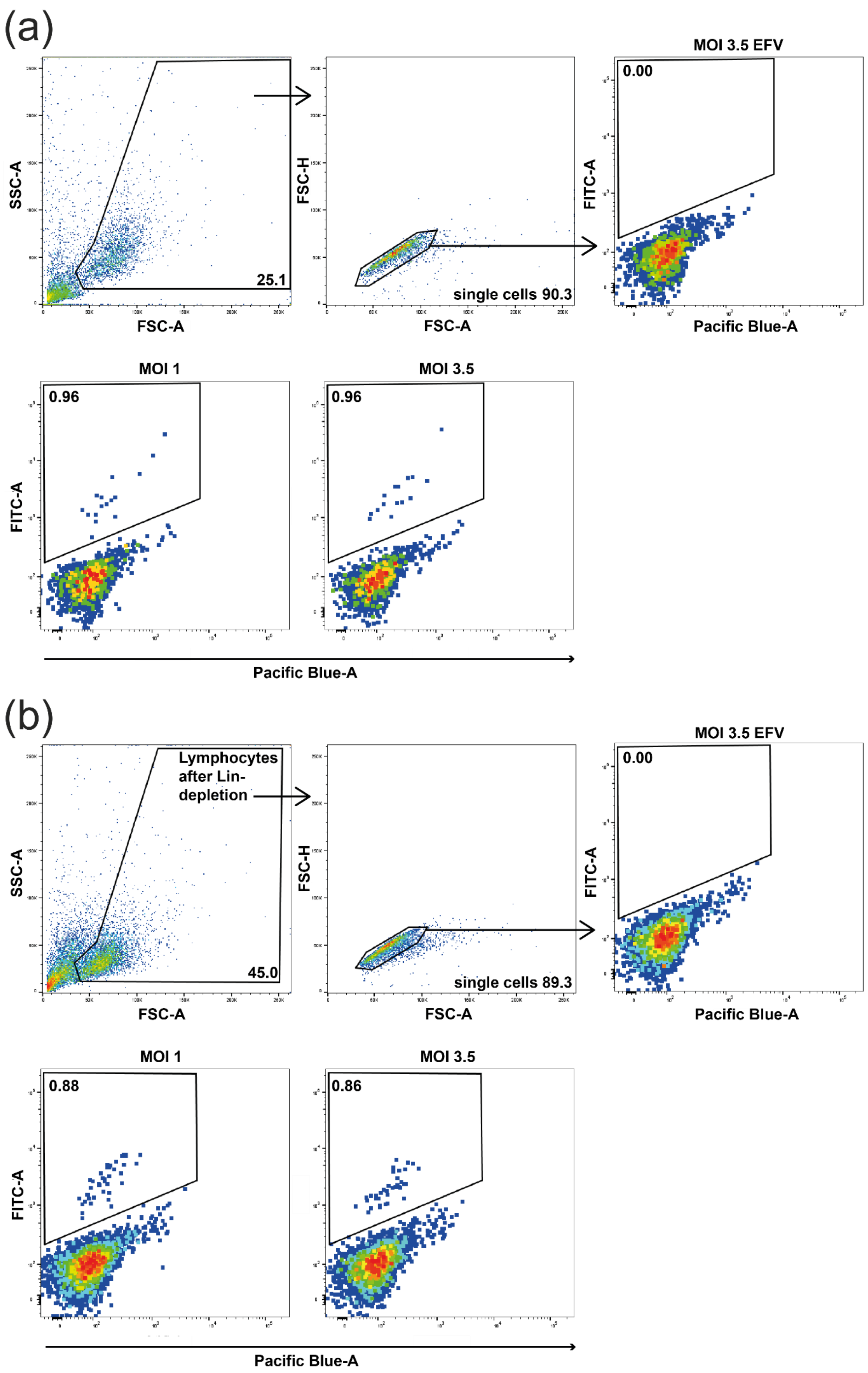

Figure S3. Flow cytometry analysis of in vitro-infected cord blood and bone marrow-derived HSPCs. (**a**,**b**) Cord blood-derived Lin- cells (A) and bone marrow derived Lin- cells (**b**) were infected with replication-competent HxB2 (X4) GFP reporter virus with an MOI of 1 and 3.5. The cells were analyzed three days after

infection for GFP expression. Depicted numbers indicate the infection rates. Cells treated with efavirenz (EFV) served as negative control. The Pacific Blue channel was used for gating only (unstained). One example of n = 3 experiments/donors is shown.

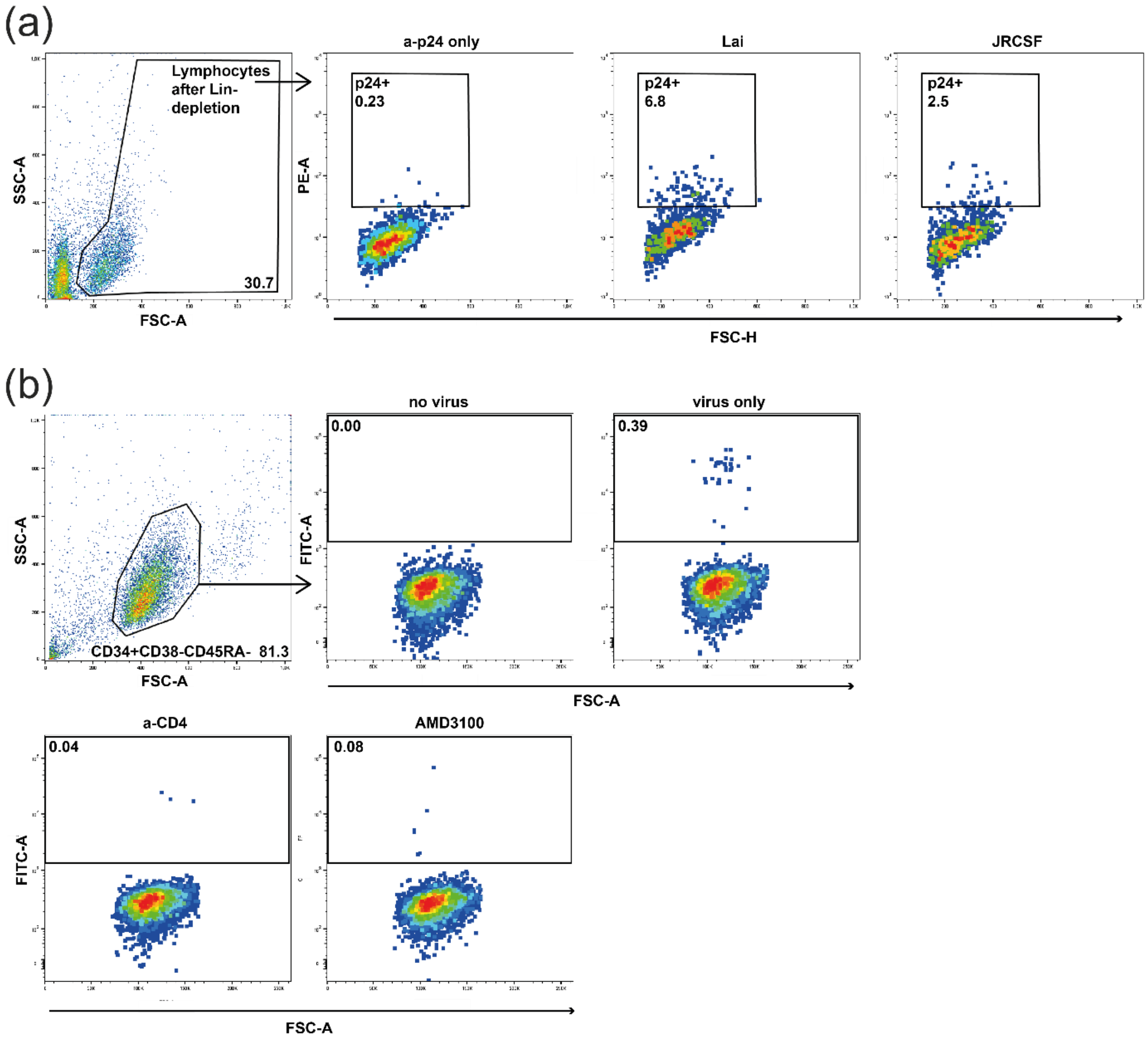

Figure S4. Flow cytometry analysis of CD4/CXCR4-dependent in vitro-infected or transduced cord blood-derived HSPCs. (**a**) cord blood-derived Lin^{-} cells infected with wild type HIV-1 Lai (X4) or JRCSF (R5); (**b**) inhibition of transduction of Lin^{-} cells using HxB2 (X4) Env-pseudotyped reporter virus with CD4 and CXCR4 inhibitors. Analysis was performed two days after infection using PE staining of p24 antigen in case of wild type HIV-1. In case of pseudotyped reporter virus transduction, GFP expression was evaluated. Numbers indicate infection/transduction rates. One example of n = 2 experiments is shown.

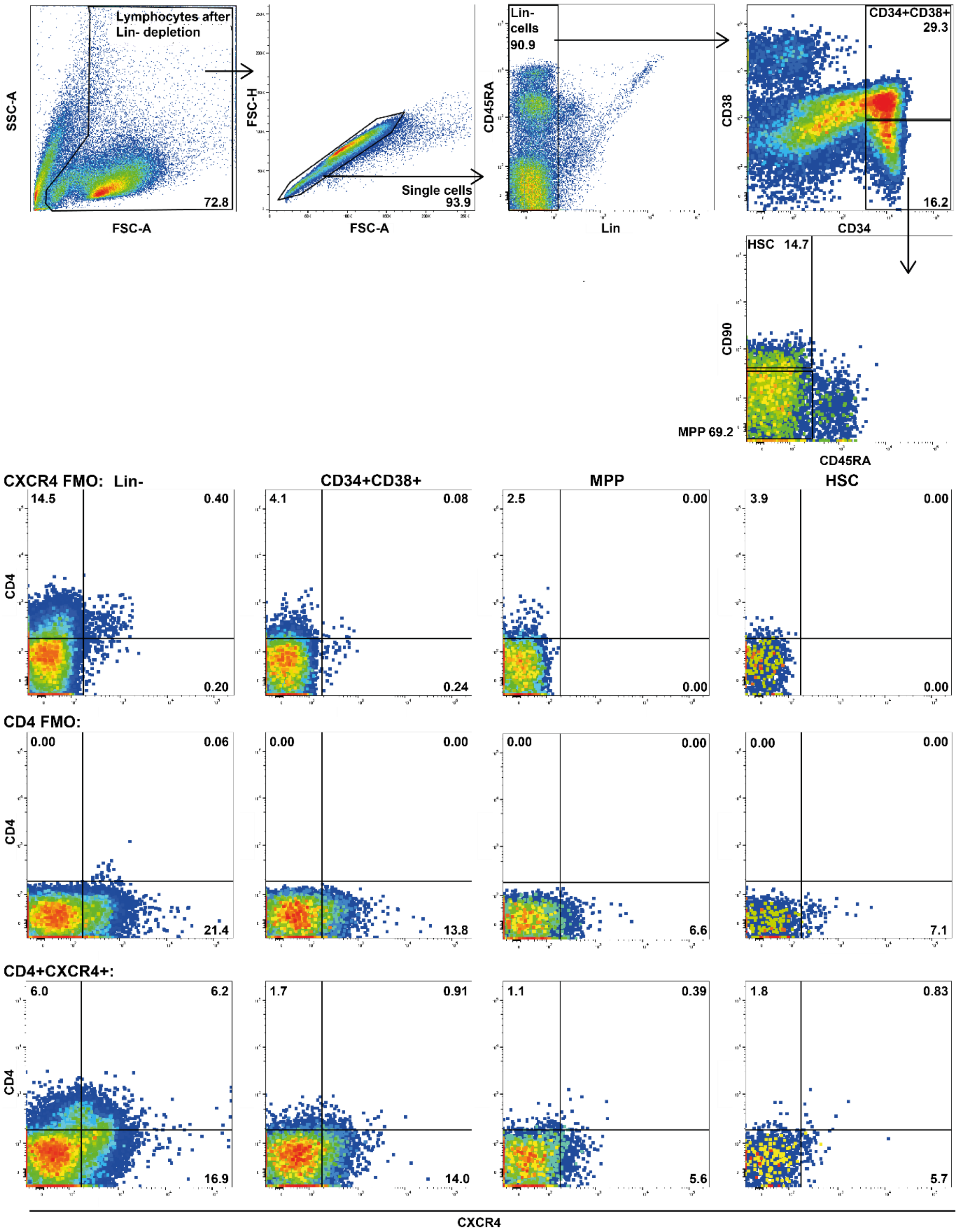

Figure S5. Flow cytometry analysis of bone marrow-derived HSPC subsets stained for CD4 and CXCR4 cell surface expression. Bone marrow-derived Lin⁻ cells were enriched by lineage depletion and subsequently stained for HSPC surface markers. The upper panel shows the gating strategy to differentiate between the HSPC subsets: CD34⁺CD38⁺: Lin⁻CD34⁺CD38⁺; multipotent progenitors (MPP): Lin⁻CD34⁺CD38⁻CD45RA⁻CD90⁻; hematopoietic stem cells (HSC): Lin⁻CD34⁺CD38⁻CD45RA⁻CD90⁺. In addition, CD4 and CXCR4 were stained to determine the amount of cells expressing HIV-1 entry receptors within the different HSPC subsets. The lower panels show the staining for the different analyzed subsets: CD4 fluorescence minus one (FMO, without α-CD4 antibody); CXCR4 FMO (without α-CXCR4 antibody); CD4/CXCR4 double stain to analyze double positive cells. One example of *n* = 9 experiments/donors is shown.

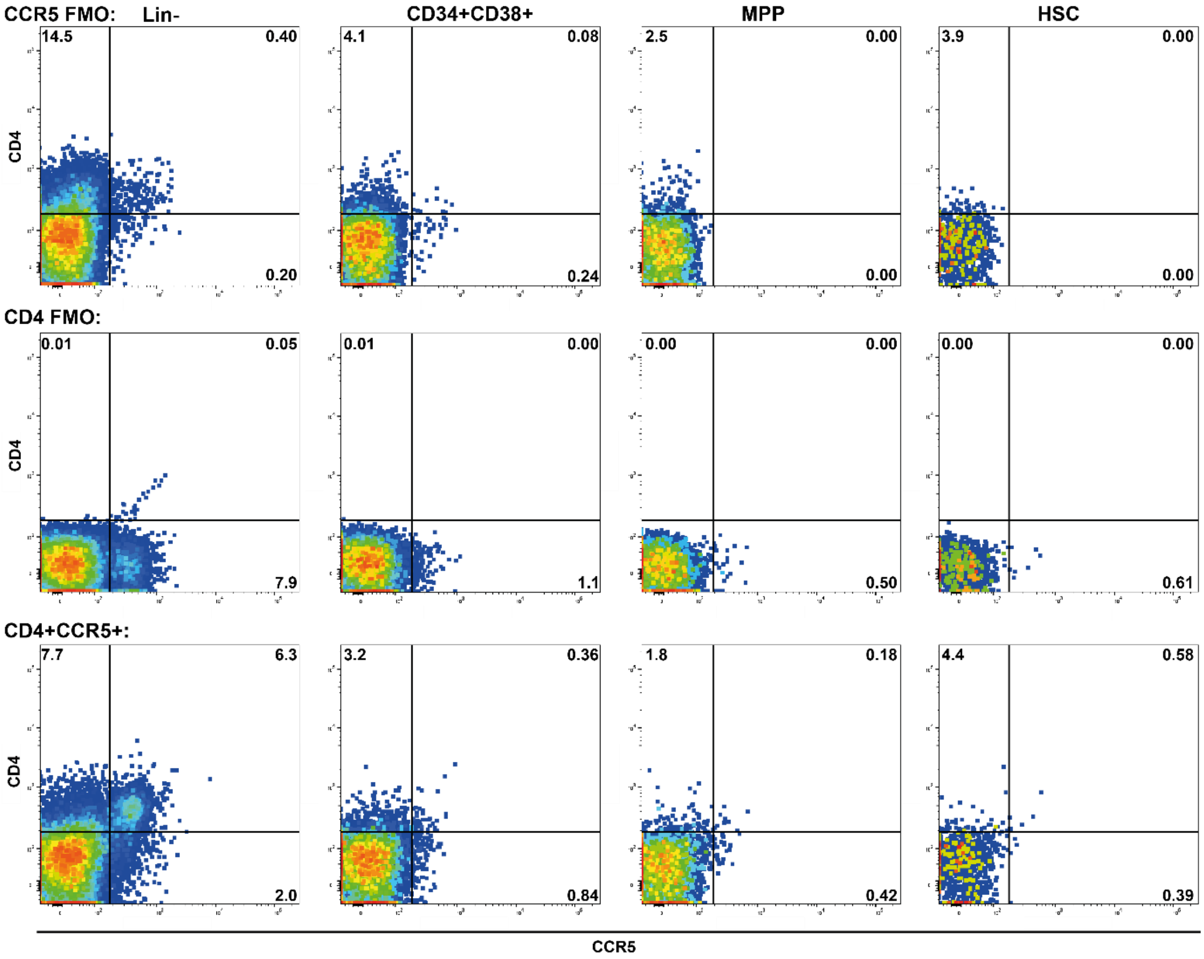

Figure S6. Flow cytometry analysis of bone marrow-derived HSPC subsets stained for CD4 and CCR5 cell surface expression. The plots show the CD4 and CCR5 staining for the different analyzed HSPC subsets: CD4 fluorescence minus one (FMO, without α-CD4 antibody); CCR5 FMO (without α-CCR5 antibody); CD4/CCR5 double stain to analyze double positive cells. One example of $n = 9$ experiments/donors is shown. MPP: Multipotent progenitors, HSC: hematopoietic stem cells.

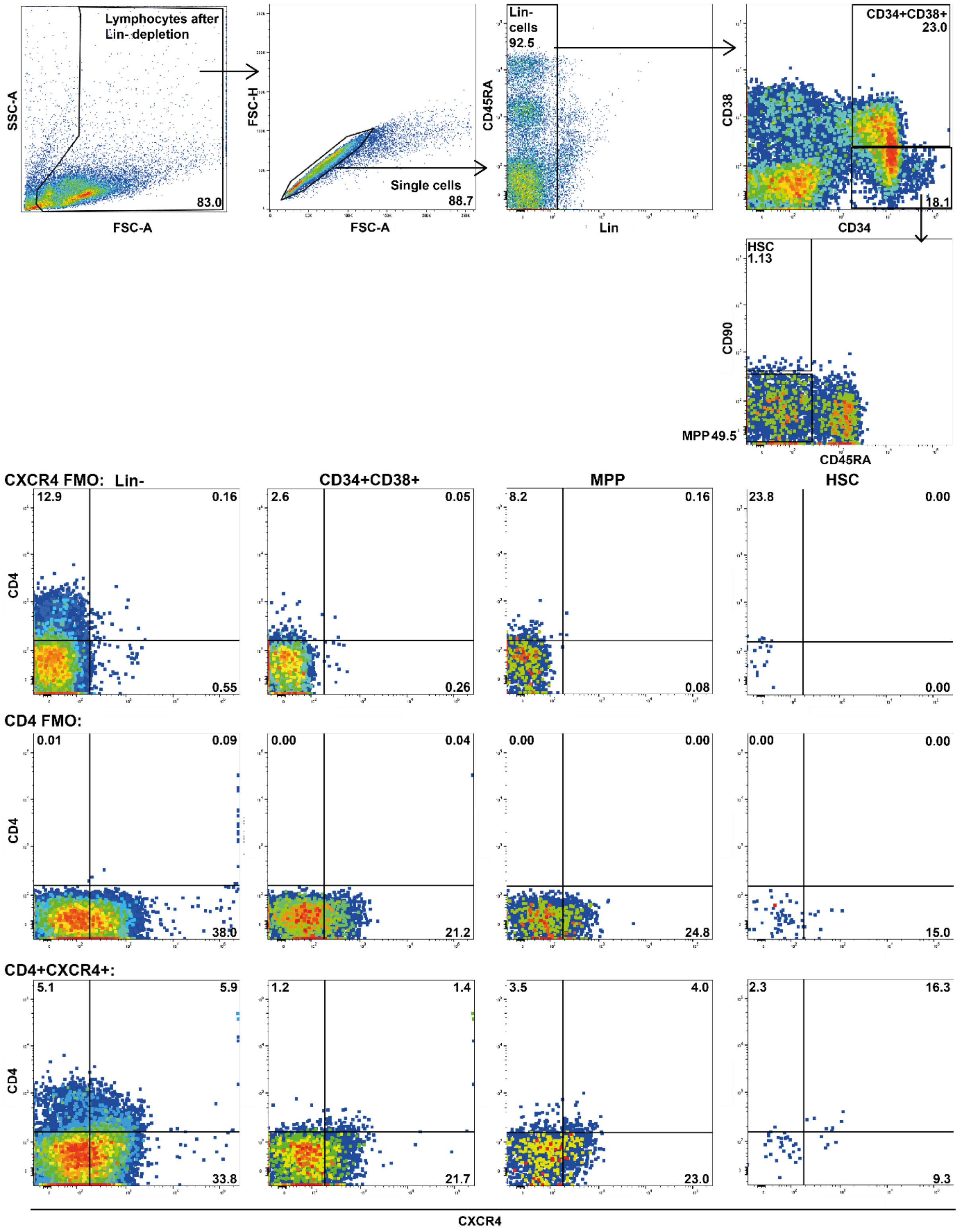

Figure S7. Flow cytometry analysis of cord blood-derived HSPC subsets stained for CD4 and CXCR4 cell surface expression. Cord blood-derived Lin$^-$ cells were enriched by lineage depletion and subsequently stained for HSPC surface markers. The upper panel shows the gating strategy to differentiate between the HSPC subsets: CD34$^+$CD38$^+$: Lin$^-$CD34$^+$CD38$^+$; multipotent progenitors (MPP): Lin$^-$CD34$^+$CD38$^-$CD45RA$^-$CD90$^-$; hematopoietic stem cells (HSC): Lin$^-$CD34$^+$CD38$^-$CD45RA$^-$CD90$^+$. In addition, CD4 and CXCR4 were stained to determine the number of cells expressing HIV-1 entry receptors within the different HSPC subsets. The lower panels show the staining for the different analyzed subsets: CD4 fluorescence minus one (FMO, without α-CD4 antibody); CXCR4 FMO (without α-CXCR4 antibody); CD4/CXCR4 double stain to analyze double positive cells. One example of n = 9 experiments/donors is shown.

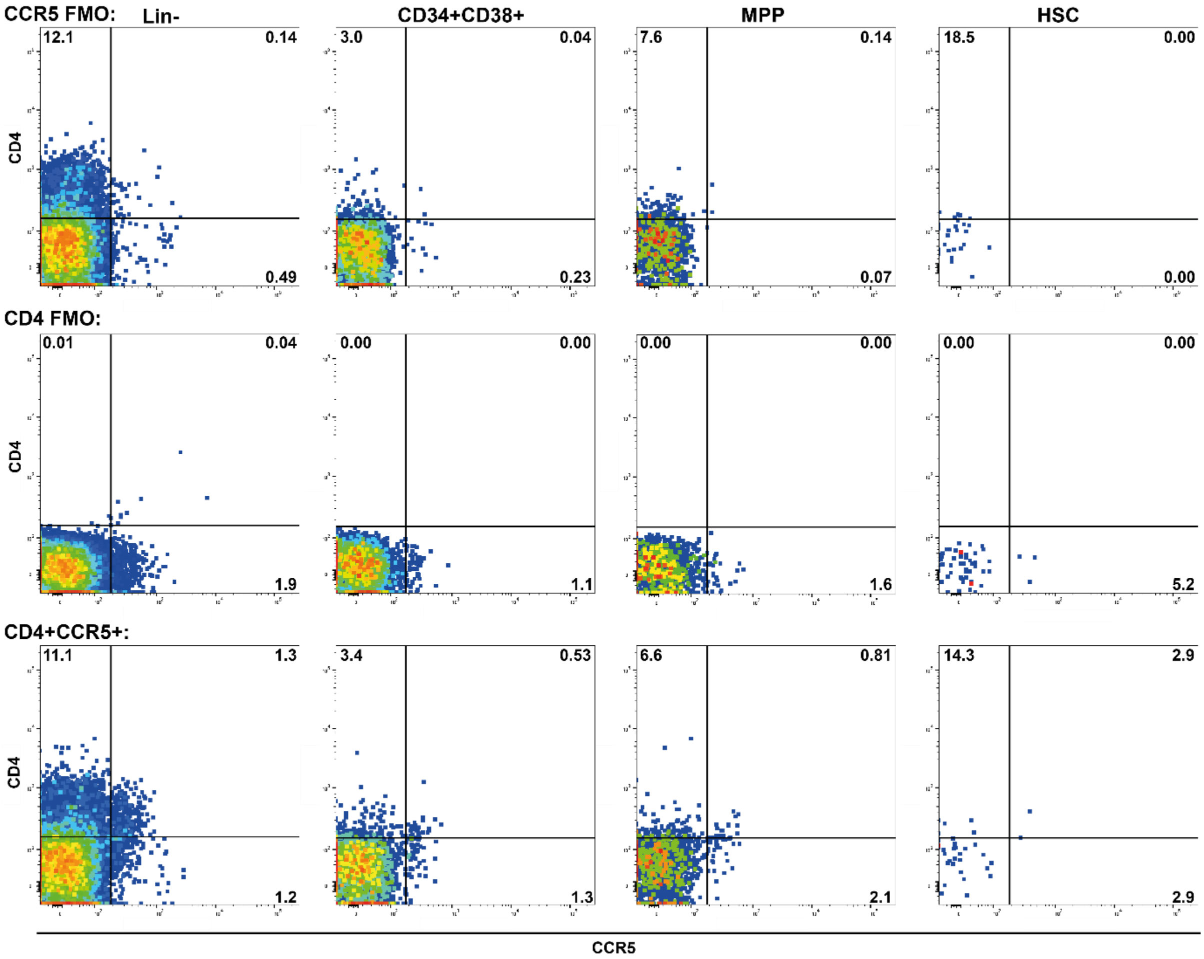

Figure S8. Flow cytometry analysis of cord blood-derived HSPC subsets stained for CD4 and CCR5 cell surface expression. The plots show the CD4 and CCR5 staining for the different analyzed HSPC subsets: CD4 fluorescence minus one (FMO, without α-CD4 antibody); CCR5 FMO (without α-CCR5 antibody); CD4/CCR5 double stain to analyze double positive cells. One example of $n = 9$ experiments/donors is shown. MPP: Multipotent progenitors, HSC: hematopoietic stem cells.

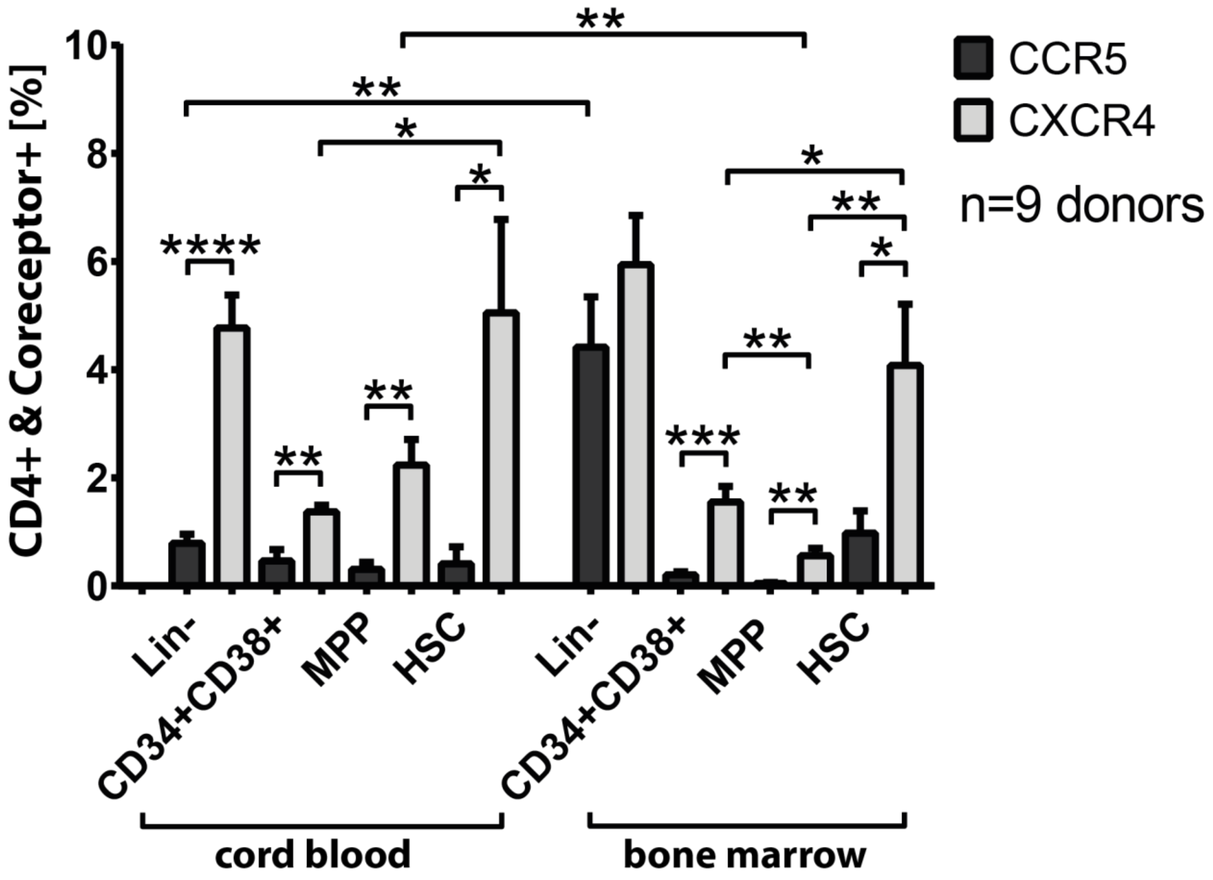

Figure S9. Frequency of CD4/CCR5 versus CD4/CXCR4 double positive cells in cord blood and bone marrow-derived hematopoietic progenitor subsets. The different HSPC subsets are Lin$^-$ cells (Lin$^+$-depleted), CD34$^+$CD38$^+$ progenitors, multipotent progenitors (MPP, CD34$^+$CD38$^-$CD45RA$^-$CD90$^-$) and hematopoietic stem cells (HSC, CD34$^+$CD38$^-$CD45RA$^-$CD90$^+$); n represents the number of different donors analyzed. Mean and standard error of the mean are indicated (* $p < 0.05$; ** $p < 0.01$; *** $p < 0.001$; **** $p < 0.0001$). Results of n = 11 experiments are shown.

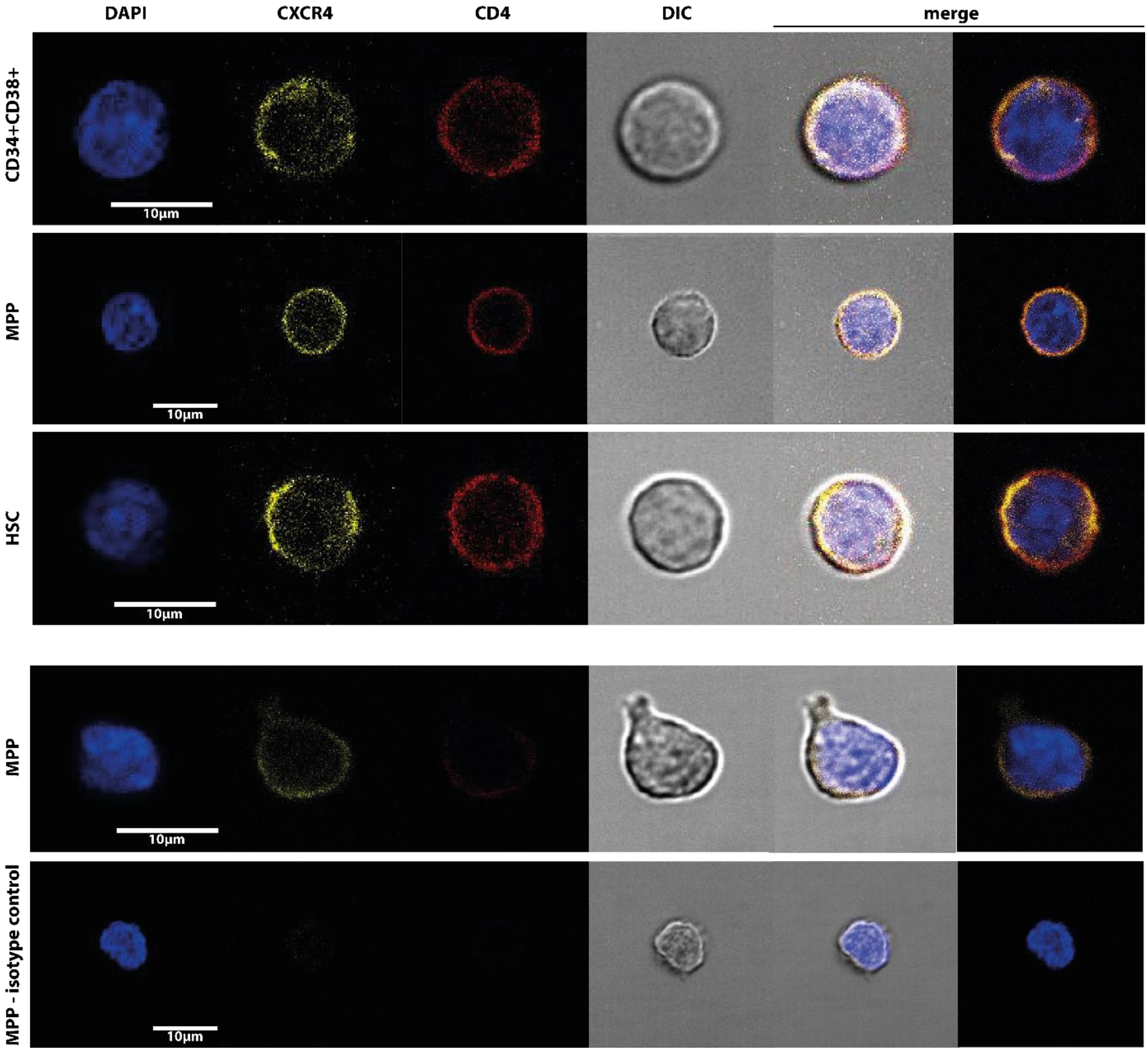

Figure S10. Confocal imaging of CD4 and CXCR4 expression in cord blood-derived hematopoietic subsets. Fluorescence confocal micrographs of different hematopoietic subsets, i.e., CD34^{+}CD38^{+} progenitors, multipotent progenitors (MPP, CD34^{+}CD38^{-}CD45RA^{-}CD90^{-}), and hematopoietic stem cells (HSC, CD34^{+}CD38^{-}CD45RA^{-}CD90^{+}). Cord blood derived Lin^{-} cells were stained for HSPC markers and sorted into CD34^{+}CD38^{+} progenitors, MPP, and HSC subsets via flow cytometry, followed by fixation, permeabilization, and staining for CD4 (red), CXCR4 (yellow), and DAPI (blue). Micrographs of the cells using separate fluorescence channels as well as differential interference contrast (DIC) and overlays of the channels (merge) are shown in a single focal plane. Upper panels show the HSPC subsets stained for CD4 and CXCR4. Lower pair of panels show MPPs stained for CD4 and CXCR4 as well as the corresponding isotype control. Representative images (of $n \geq 30$ images) of CXCR4^{+}CD4^{+} double-positive cells among the mixed population of CXCR4^{-}CD4^{-} (relative abundance ≤ 5%), CXCR4^{+}CD4^{-} (relative abundance ≥ 60%), and CXCR4^{+}CD4^{+} cells in respective HSPC subsets in $n = 3$ experiments are shown. Differentiated cells were eliminated during lineage depletion and sort, and the likeliness of contaminating CD3^{+} T cells is below 0.3% (1% and 3% maximum impurity after lineage depletion and sort, respectively, Figure S1).

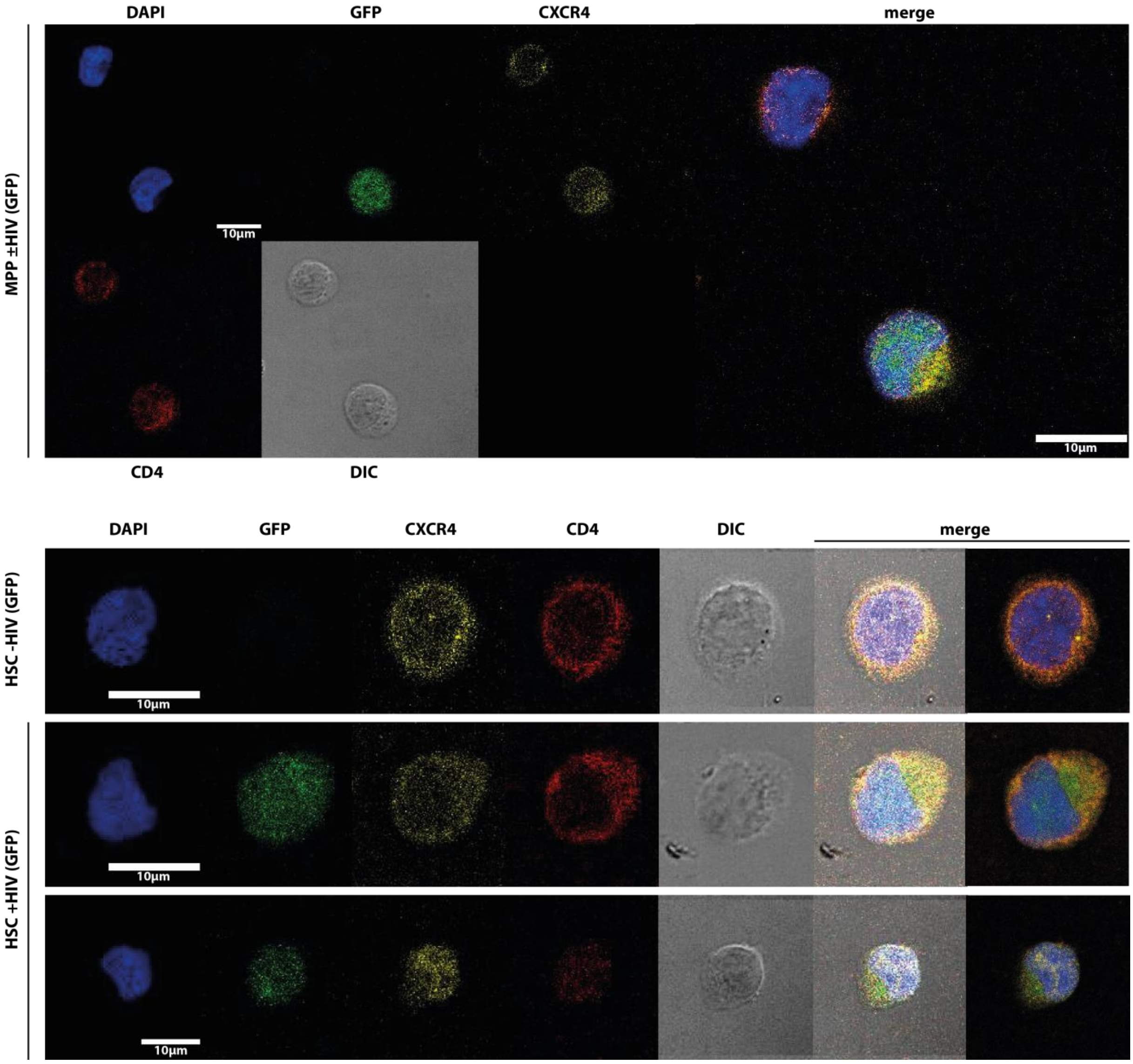

Figure S11. Confocal imaging of CD4 and CXCR4 expression in HSPCs transduced with HXB2 Env-pseudotyped GFP reporter virus. Fluorescence confocal micrographs of transduced (GFP$^+$) and non-transduced (GFP$^-$), cord blood-derived multipotent progenitors (MPP, CD34$^+$CD38$^-$CD45RA$^-$CD90$^-$) and hematopoietic stem cells (HSC, CD34$^+$CD38$^-$CD45RA$^-$CD90$^+$). Cord blood-derived Lin$^-$ cells were stained for HSPC markers, sorted into MPP and HSC subsets followed by transduction using HXB2 Env-pseudotyped GFP reporter virus (MOI = 10). Fixation, permeabilization and staining of CXCR4 (yellow), CD4 (red), and DAPI (blue) was performed five days post transduction. Micrographs of the cells using separate fluorescence channels, differential interference contrast (DIC), and overlays of the channels (merge) are shown in a single focal plane. Representative images (of $n \geq 5$ images per HSPC subset) of GFP$^+$CXCR4$^+$CD4$^+$ cells among the mixed population of GFP$^-$CXCR4$^{+/-}$CD4$^{+/-}$ cells (relative abundance >95%) and GFP$^+$CXCR4$^+$CD4$^+$ cells in the indicated HSPC subsets in $n = 2$ experiments. Differentiated cells were eliminated during lineage depletion and sort, and the likeliness of contaminating CD3$^+$ T cells is below 0.3% (1% and 3% maximum impurity after lineage depletion and sort, respectively, Figure S1).

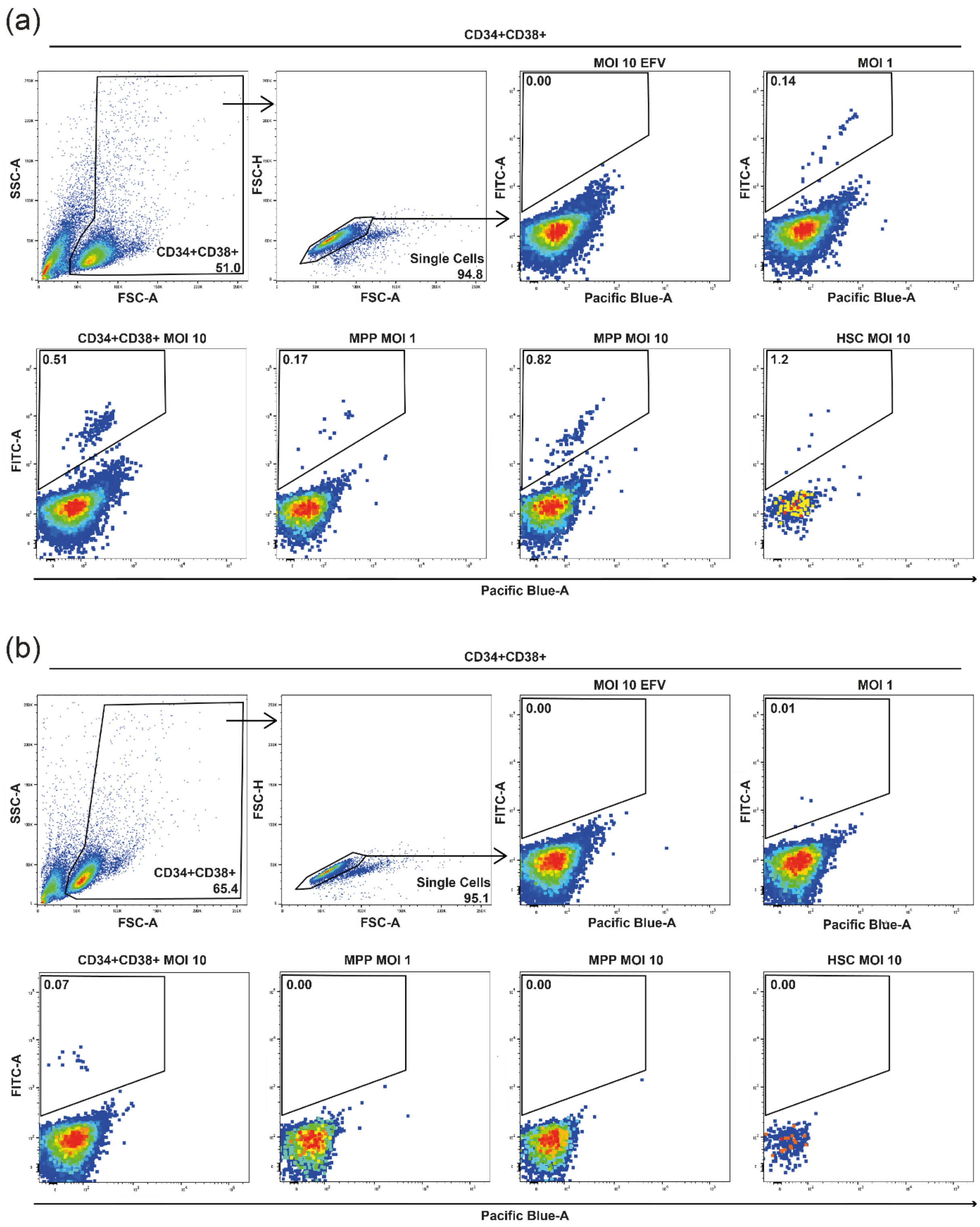

Figure S12. Flow cytometry analysis of in vitro-transduced bone marrow-derived HSPC subsets. (**a**,**b**) Bone marrow-derived HSPC subsets were transduced with HxB2 Env (**a**) or JRFL Env (**b**)-pseudotyped reporter viruses with an MOI of 1 and 10. The cells were analyzed three days after transduction for GFP expression. Depicted numbers indicate the infection rates. Cells treated with efavirenz (EFV) served as negative control. The Pacific Blue channel was used for gating only (unstained). CD34$^+$CD38$^+$: Lin$^-$CD34$^+$CD38$^+$; multipotent progenitors (MPP): Lin$^-$CD34$^+$CD38$^-$CD45RA$^-$CD90$^-$; hematopoietic stem cells (HSC): Lin$^-$CD34$^+$CD38$^-$CD45RA$^-$CD90$^+$. One representative example of $n = 6$ (**a**) and $n = 2$ (**b**) experiments/donors, respectively is shown.

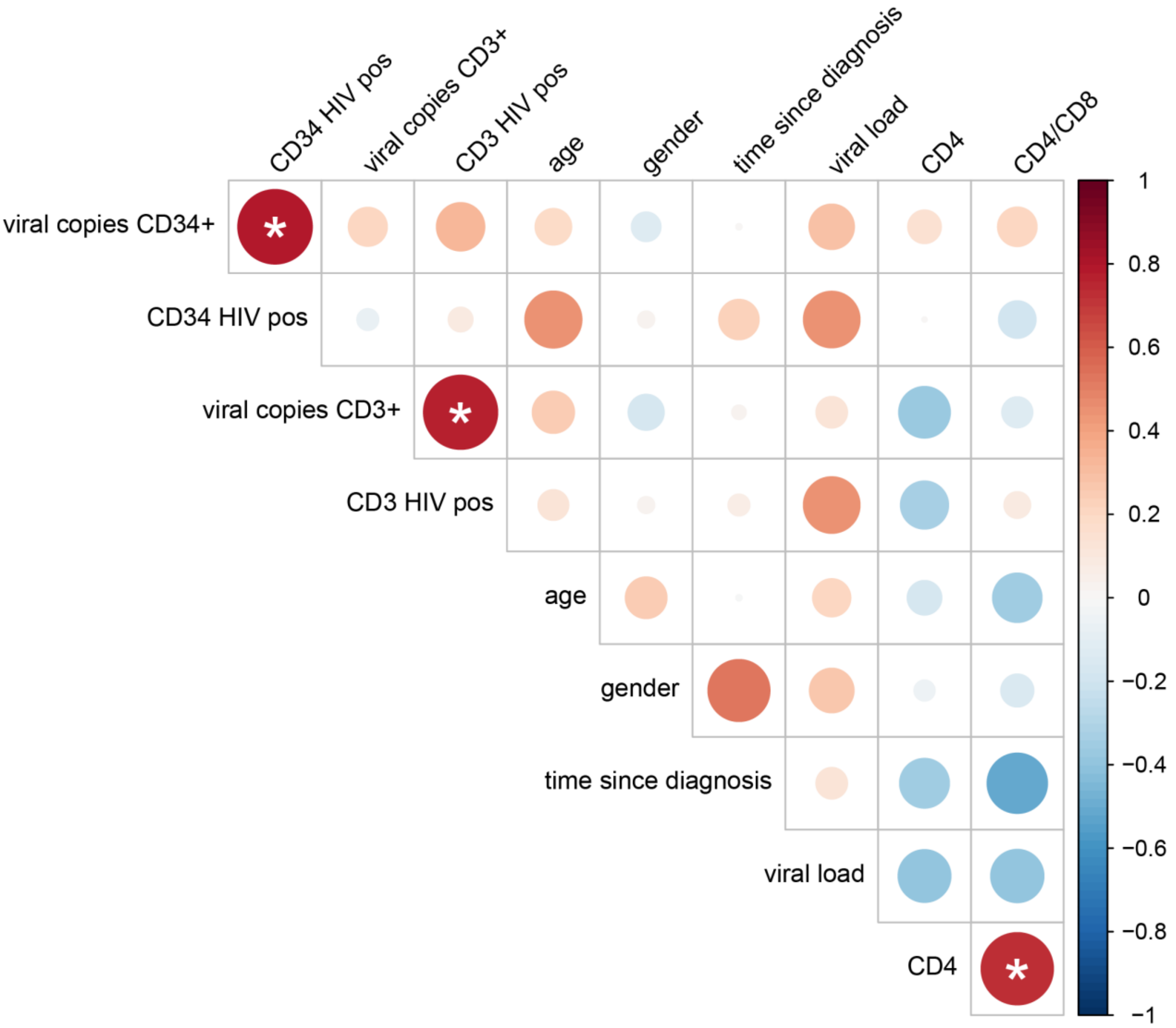

Figure S13. Correlation analysis of qPCR, patient, and clinical data of donors screened for CD34^{+} and CD3^{+} infected cells (Source: Table S2). Correlogram with circles, sized and color-coded according to linear regression coefficients (*r*) of correlations between the indicated variables. A color scheme indicating *r* values is provided to the right (red for positive correlations, blue for inverse correlations). Asterisks indicate statistically significant correlations. CD34 HIV pos and CD3 HIV pos: discrete discrimination of positive (value =1) or absent detection (value = 0) of HIV DNA in Lin^{-}CD34^{+} or CD3^{+} cells, respectively; CD4/CD8: ratio of CD4^{+} and CD8^{+} T cells; # cells: number of cells.

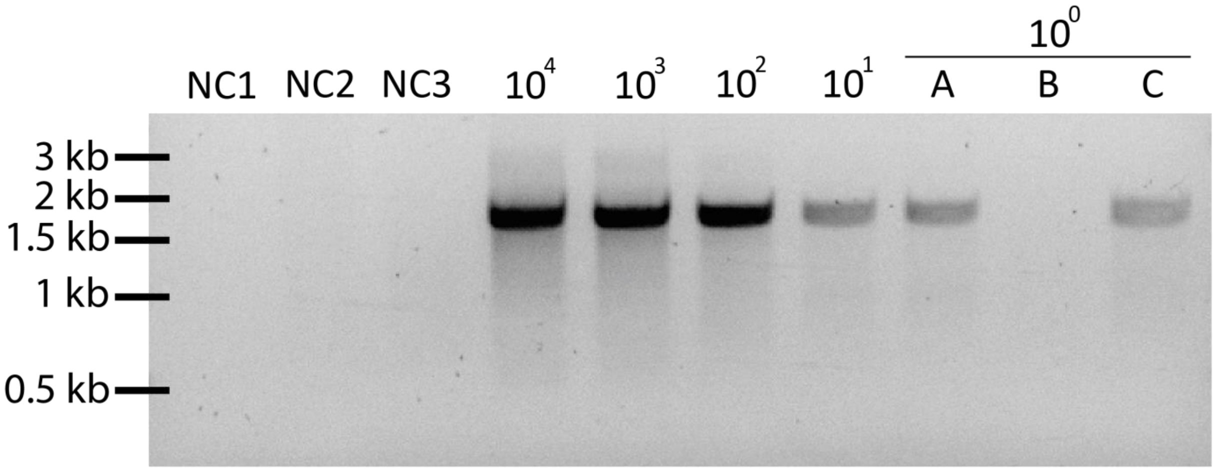

Figure S14. Sensitivity of nested *env* PCR using endpoint dilution. The sensitivity of the nested *env* PCR was determined by serial dilution of ACH-2 cells, harboring a single copy of the latently integrated HIV-1 Lai genome, in uninfected PM1 cells using a total of 1–1.1×10^5 cells. The numbers of ACH-2 cells present in the respective dilutions are indicated on top. A 1% agarose gel of the nested PCR products after the second round PCR is shown. The applied samples are as follows: NC1 = water control 2nd run, NC2 = water control 1st run, NC3 = 10^5 PM1 cells, dilutions containing 10^4, 10^3, 10^2, 10^1 and 10^0 (A, B, and C represent 3 independent dilutions) ACH-2 cells. The experiment was performed once.

Table S1. Comparison of HSPC transduction rates with X4 and R5 HIV-1 Env-pseudotyped reporter viruses. The data refers to the results shown in Figures 1a and 2. Cord blood and bone marrow-derived lineage-depleted (Lin^-) cells (upper section) and bone marrow-derived $CD34^+CD38^+$ progenitors (lower section) were transduced using replication incompetent HxB2 (X4) and JRFL (R5) HIV-1 Env-pseudotyped GFP reporter virus with MOIs of 1 and 10. The percentage [%] of GFP-positive cells was determined 72 h post-transduction. For each experiment, means and standard errors of the mean (in parenthesis) are indicated. *p*-values (*p*) were calculated using an unpaired, two-tailed *t*-test comparing R5-tropic and X4-tropic transduction events. Data from $n = 6$ (X4), $n = 3$ (R5; both Figure 1A), and for $n = 6$ (X4) and $n = 2$ (R5; both Figure 2) similar experiments are shown.

	**X4 vs R5 Transduction Rates (GFP^+)**					
**Cell subset/MOI**	**Lin^-/MOI1**			**Lin^-/MOI10**		
	**X4**	**R5**	**X4 vs R5 (p)**	**X4**	**R5**	**X4 vs R5 (*p*)**
Cord blood (Figure 1A)	0.172 (0.091)	0.007 (0.007)	0.253	0.629 (0.202)	0.008 (0.008)	0.074
Bone marrow (Figure 1A)	0.080 (0.027)	0.011 (0.011)	0.133	0.351 (0.115)	0.019 (0.019)	0.901
**Cell subset/MOI**	**$CD34^+CD38^+$/MOI1**			**$CD34^+CD38^+$/MOI10**		
	**X4**	**R5**	**X4 vs R5 (*p*)**	**X4**	**R5**	**X4 vs R5 (p)**
Bone marrow (Figure 2)	0.217 (0.062)	0.012 (0.004)	0.059	0.769 (0.145)	0.063 (0.014)	0.015

Table S2. Comparison of HSPC transduction rates with X4 and R5 HIV-1 Env-pseudotyped reporter viruses for individual donors**.** The data refers to the results shown in Figure 1a. Donors are shown from whom specimens were studied for both HxB2 (X4) and JRFL (R5) HIV-1 Env-pseudotyped GFP reporter virus transduction. Cord blood and bone marrow-derived lineage-depleted (Lin^-) cells were transduced using replication incompetent HxB2 (X4) and JRFL (R5) HIV-1 Env-pseudotyped GFP reporter virus with MOIs of 1 and 10. The percentage (%) of GFP-positive cells was determined 72 h post-transduction. Means are indicated.

	**HXB2**				**JRFL**			
	**Uninfected**	**EFV control**	**MOI of 1**	**MOI of 10**	**Uninfected**	**EFV control**	**MOI of 1**	**MOI of 10**
**CB008**	0.0%	0.0%	0.0%	0.816%	0.0%	0.0%	0.020%	0.023%
**BMHI014**	0.0%	0.023%	0.086%	0.207%	0.0%	0.0%	0.034%	0.0%
**BMHI015**	0.0%	0.0%	0.119%	0.512%	0.0%	0.0%	0.0%	0.056%
**BMHI017**	0.0%	0.0%	0.0%	0.050%	0.0%	0.0%	0.0%	0.0%

Table S3. Demographic and clinical data of study participants. Clinical data of donors screened by qPCR for $CD34^+$ and $CD3^+$ infected cells. Units are of age in years at bone marrow aspiration (Age). Time since diagnosis is in years since diagnosis at the time point of bone marrow aspiration. Viral load is measured in RNA copies/ml serum. $CD4^+$ cells are measured in cells/µL; $CD3^+$ and $CD34^+$ cells are measured as number of cells isolated and tested per individual. The blue background indicates a positive detection of proviral HIV genomes according to qPCR results in Figure 6; n.d.: not determined.

Code	Age	Sex	Time Since Diagnosis	Viral Load	CD4	CD4/CD8	Therapy	Subtype	Tropism	$CD3^+$ Cells	$CD34^+$ Cells
BM001	51	M	0.0	60300	186	0,3	Kivexa, Isentress	B	R5	3×10^6	1.2×10^6
BM002	27	M	1.0	<20	502	0,42	Truvada, Kaletra	n.d.	n.d.	3.7×10^6	6.5×10^5
BM003	36	M	0.0	<20	382	6,91	Truvada, Reyataz, Norvir	B	R5	2×10^5	2×10^4
BM004	57	F	3.2	<20	696	0,59	Kaletra	n.d.	n.d.	4×10^6	7×10^5
BM005	47	M	10.3	<20	1351	0,98	Truvada, Telzir, Norvir	n.d.	R5	6×10^5	5×10^4
BM006	59	M	3.8	36	66	0,14	Truvada, Reyataz, Norvir	B	R5	3.5×10^6	1×10^6
BM007	52	F	4.8	3590	394	0,41	Isentress, Prezista, Norvir	CRF02_AG	R5	2.9×10^6	2.4×10^5
BM008	33	F	17.0	195000	114	0,3	Truvada, Prezista, Norvir	B	n.d.	2.1×10^6	2×10^4
BM009	47	M	3.1	<20	274	2,55	Truvada, Prezista, Norvir	n.d.	R5	1×10^5	3×10^4
BM0010	47	M	12.1	<20	177	0,14	Truvada, Retrovir	n.d.	R5	7.4×10^5	1.4×10^5
BM0011	50	W	12.5	<20	95	0,24	Truvada, Intelence	D	n.d.	3.4×10^4	1.5×10^4
